# Retraction: Silencing of Prrx1b suppresses cellular proliferation, migration, invasion and epithelial–mesenchymal transition in triple‐negative breast cancer

**DOI:** 10.1111/jcmm.18312

**Published:** 2024-04-25

## Abstract

Zhi‐Dong Lv, Zhao‐Chuan Yang, Xiang‐Ping Liu, Li‐Ying Jin, Qian Dong, Hui‐Li Qu, Fu‐Nian Li, Bin Kong, Jiao Sun, Jiao‐Jiao Zhao, Hai‐Bo Wang, Silencing of Prrx1b suppresses cellular proliferation, migration, invasion and epithelial–mesenchymal transition in triple‐negative breast cancer. *Journal of Cellular and Molecular Medicine*, 20: 1640–1650. https://doi.org/10.1111/jcmm.12856

The above article, published online on 29 March 2016 in Wiley Online Library (wileyonlinelibrary.com), has been retracted by agreement between the authors, the journal Editor‐in‐Chief, Stefan Constantinescu, The Foundation for Cellular and Molecular Medicine and John Wiley and Sons Ltd. In 2022, the authors asked to retract the article as they found that some images were mistakenly duplicated from their previously published papers. Subsequently, an investigation into concerns raised by a third party, revealed inappropriate duplications of image panels within the article (Figures 2A,B and 4D) as well as between this (Figure 1B) and three other articles (with the same first or corresponding author) that were published previously in a different scientific context. Due to the number and the level of errors identified in the published figures, the editors consider the conclusions of this manuscript substantially compromised. Although the authors initially asked for the retraction, they did not agree to the retraction wording.

Zhi‐Dong Lv, Zhao‐Chuan Yang, Xiang‐Ping Liu, Li‐Ying Jin, Qian Dong, Hui‐Li Qu, Fu‐Nian Li, Bin Kong, Jiao Sun, Jiao‐Jiao Zhao, Hai‐Bo Wang, Silencing of Prrx1b suppresses cellular proliferation, migration, invasion and epithelial–mesenchymal transition in triple‐negative breast cancer. *Journal of Cellular and Molecular Medicine*, 20: 1640–1650. https://doi.org/10.1111/jcmm.12856

The above article, published online on 29 March 2016 in Wiley Online Library (wileyonlinelibrary.com), has been retracted by agreement between the authors, the journal Editor‐in‐Chief, Stefan Constantinescu, The Foundation for Cellular and Molecular Medicine and John Wiley and Sons Ltd. In 2022, the authors asked to retract the article as they found that some images were mistakenly duplicated from their previously published papers. Subsequently, an investigation into concerns raised by a third party, revealed inappropriate duplications of image panels within the article (Figures 2A,B and 4D) as well as between this (Figure 1B) and three other articles (with the same first or corresponding author) that were published previously in a different scientific context. Due to the number and the level of errors identified in the published figures, the editors consider the conclusions of this manuscript substantially compromised. Although the authors initially asked for the retraction, they did not agree to the retraction wording.

